# Digital Light Processing Route for 3D Printing of Acrylate-Modified PLA/Lignin Blends: Microstructure and Mechanical Performance

**DOI:** 10.3390/polym16101342

**Published:** 2024-05-09

**Authors:** Sofiane Guessasma, Nicolas Stephant, Sylvie Durand, Sofiane Belhabib

**Affiliations:** 1 INRAE, Research Unit BIA UR1268, Rue Geraudiere, F-44316 Nantes, France; sylvie.durand@inrae.fr; 2 Nantes Université, CNRS, Institut des Matériaux Jean Rouxel, IMN, F-44300 Nantes, France; nicolas.stephant@univ-nantes.fr; 3 Department of Mechanical Engineering, Nantes Université, IUT, 2 Av. du Professeur Jean Rouxel, F-44470 Carquefou, France; sofiane.belhabib@univ-nantes.fr

**Keywords:** digital light processing, photosensitive polylactic acid, lignin, tensile performance, X-ray microtomography

## Abstract

In this study, digital light processing (DLP) was utilized to generate 3D-printed blends composed of photosensitive acrylate-modified polylactic acid (PLA) resin mixed with varying weight ratios of lignin extracted from softwood, typically ranging from 5 wt% to 30 wt%. The microstructure of these 3D-printed blends was examined through X-ray microtomography. Additionally, the tensile mechanical properties of all blends were assessed in relation to the weight ratio and post-curing treatment. The results suggest that post-curing significantly influences the tensile properties of the 3D-printed composites, especially in modulating the brittleness of the prints. Furthermore, an optimal weight ratio was identified to be around 5 wt%, beyond which UV light photopolymerization experiences compromises. These findings regarding acrylate-modified PLA/lignin blends offer a cost-effective alternative for producing 3D-printed bio-sourced components, maintaining technical performance in reasonable-cost, low-temperature 3D printing, and with a low environmental footprint.

## 1. Introduction 

Additive manufacturing (AM) has emerged as a revolutionary technology that has attracted considerable attention in recent decades [1,2,3]. It holds tremendous potential as a processing technology for crafting intricately complex technical components [4,5]. A widely accepted definition of AM is the methodical deposition of material layer by layer based on a digitalized model [6,7]. A distinctive feature of AM is its ability to locally control material deposition [5,8], allowing for complete customization of parts with minimal reliance on tooling [9,10]. This ability also enables the creation of innovative material generations, such as adaptive materials [11]. The inherent rapid fabrication cycle in AM makes it an attractive technology for various sectors, including bioengineering, aeronautics, civil engineering, prototyping, automotive, engineering, the food industry, and art, among others [12,13,14,15,16,17,18,19]. The widespread adoption of AM in multiple industries is attributed to the diverse processes falling within its definition, enabling the printing of a wide range of materials [3,20]. For instance, FFF (fused filament fabrication) or FDM (fused deposition modeling) are popular and cost-effective methods for printing polymeric structures [21,22,23]. Laser-based technologies, like SLM (selective laser melting), are used for handling metallic powders [24,25,26]. Stereolithography, another process employing a laser source, targets photosensitive resins [27,28]. This technology, known for its high-resolution processing compared to fused filament, produces highly isotropic structures [29]. Stereolithography operates by having a laser beam scan the surface of a photosensitive resin in a liquid state [27,28]. The polymerization of the structure occurs at the spot targeted by the laser beam based on the 2D pattern derived from the slicing step [27,30]. Although stereolithography achieves high resolution, its extended process time is a drawback due to the extensive toolpath scanning [31]. Digital light processing (DLP) is another AM method that shares the rationale of stereolithography but addresses the time-consuming scanning issue [32]. DLP utilizes a digital screen to project an image of the layer under construction, enabling the polymerization of an entire layer instead of a single spot [33,34]. Products based on DLP are primarily used for prototyping, molding, and consumer products [33,35]. Salih et al. [36] considered the use of DLP to process composite materials using acrylonitrile butadiene styrene (ABS) as a matrix and carbon fibers as reinforcement. Zhao et al. [37] considered the same process to develop Kevlar composites. These achievements deal with the use of a synthetic material. In this study, we propose to valorize a biomass through lignin, which was mainly considered a feedstock material for energy applications. Our purpose is to improve the environmental footprint of photosensitive resins and evaluate the potential of lignin as a substitute in DLP technology. 

In this research, we introduce, for the first time, a successful endeavor to process a bio-sourced blend comprising lignin extracted from softwood and acrylate-modified PLA through the photopolymerization route. This route was selected in this work to print this polymer to avoid the degradation of lignin which cannot withstand high extrusion temperatures in FDM or SLM. Lignin functions as a solid filler in a photosensitive acrylate-modified PLA resin, and we delve into both the processing conditions and the quantity involved. Additionally, we explore the assessment of composite performance and microstructural features. The expensive nature of photosensitive resins traditionally constrains material options for processing. Blending provides a remedy for designing structures with specific performance attributes, such as water sorption/desorption capability, while maintaining reasonable printing costs. Furthermore, the utilization of biomass aligns with the current trend in the plastics industry, given its favorable environmental footprint, high specific mechanical properties, biocompatibility, and transfer properties. For example, Azmin et al. [38] formulated bioplastic films from agricultural wastes, showcasing low water adsorption and vapor permeability. Fatima et al. [39] developed non-toxic biocomposite materials using bacterial cellulose for biomedical applications, highlighting their potential in transfer properties while addressing environmental and economic concerns. This investigation establishes that lignin is a viable option, preserving the resin’s performance and presenting favorable mechanical properties. Another impetus for this research is to address the challenges associated with processing lignin using alternative AM methods. Processing lignin proves challenging in FDM, requiring a functionalization step to blend it beyond the glass transition. Indeed, the lack of compatibility would result in a significant decline in mechanical performance, making it unfeasible to support layering during the printing process. In this study we explore the use of lignin as a bio-sourced material to lower the environmental footprint of photosensitive resins used in the manufacturing of technical parts through DLP technology. In addition, the cost reduction and optimal lignin contents are also points of focus of this study. 

## 2. Experimental Layout

### 2.1. Process and Materials 

**Figure 1:**
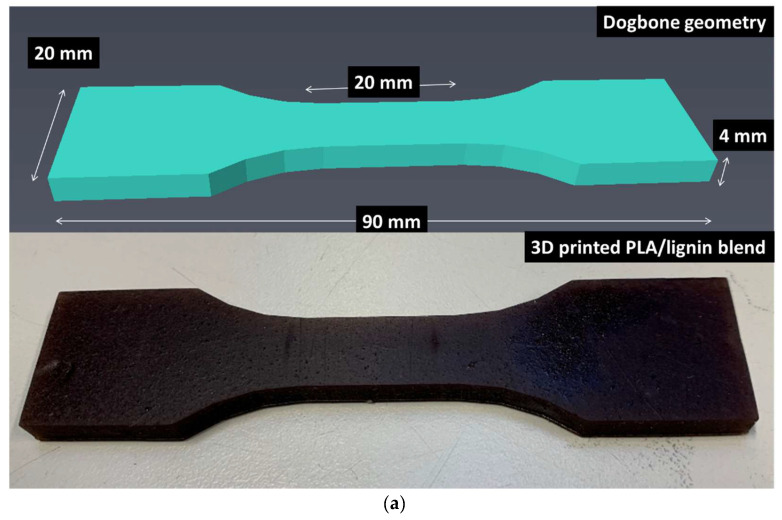

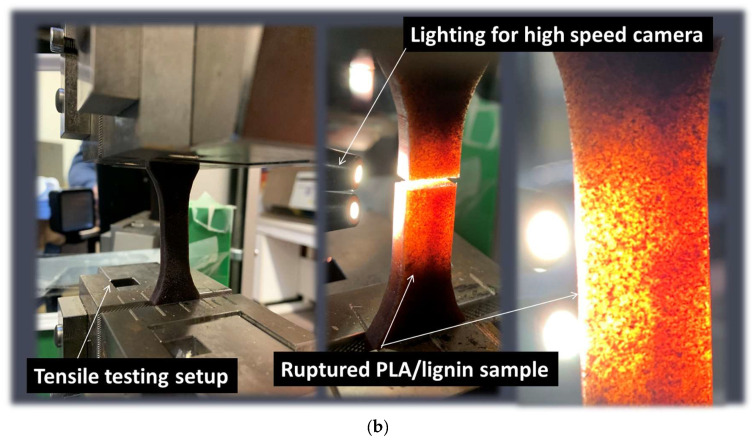
Overview of the geometry and testing of DLP samples: (**a**) acrylate-modified PLA/lignin geometry and printed part, (**b**) tensile testing experiment under optical recording.

The DLP 3D printer employed in this research features was an Elegoo Mars (Elegoo France, Lespinasse, France) having a build space measuring 120 × 68 × 155 mm^3^ (Figure 1a). The resolution of the equipment is intricately linked to the pixel size of the LED screen, which measures approximately 50 µm. The achieved resolution is 2560 × 1440 pixels, and the accuracy in axis positioning is 1.25 µm.

The feedstock materials utilized in this study include an acrylate-modified PLA-like photosensitive resin obtained from e-sun 3D France (Lespinasse, France). Table 1 outlines the primary characteristics of the acrylate-modified PLA photosensitive resin. 

This resin is composed of acrylic compounds, encompassing polymerizable unsaturated monomers such as butyl, ethyl, and methyl acrylate, a polymeric binder made of polybutadiene, polymerization initiators represented by benzoin, and a thermal polymerization inhibitor, hydroquinone. The second component is acrylate-modified PLA. The filler comprises lignin from soft wood purchased from Borregaard (Sarpsborg, Norway), characterized by a typical granulometry below 100 µm.

The weight percentage of lignin ranges from 5 wt% to 30 wt%. Dog-bone specimens, measuring 90 × 20 × 4 mm^3^ with a gauge length of 20 mm and a gauge width of 10 mm, were fabricated using UV light with a wavelength of 405 nm (Figure 1a). The exposure time was set at 20 s for the initial five layers and 7 s for the subsequent layers. The entire printing process took 15 min. Subsequently, the specimens underwent a post-curing step involving an additional 15 min exposure to UV light after reaching the green state. The equipment used for post-curing was a standard 4-lamp 36W UV 320–400 nm device from HSNF Ltd. (London, UK). 

### 2.2. Characterization Techniques

The mid-infrared spectroscopy analysis of both acrylate-modified PLA and acrylate-modified PLA-like photosensitive resins was conducted using a Thermo Nicolet IS50 spectrometer (Thermo Scientific, Courtaboeuf, France). Spectra were collected in reflection mode spanning from 4000 to 400 cm^−1^ at a resolution of 8 cm^−1^, employing a Smart iTX—ATR diamond accessory. The infrared spectra were derived from 200 co-added scans using OMNIC 9.2.41 software. Subsequently, all spectra within the 4000–700 cm^−1^ range were subjected to baseline correction and unit vector normalization. Mean spectra were generated from three repetitions using OPUS 7.5 software (Bruker France SAS, Champs-sur-Marne, France). By comparing these spectra with those available in the OPUS library, the primary components present in the resin were identified.

Mechanical testing of 3D-printed acrylate-modified PLA/lignin specimens was conducted using a Zwick Roell universal machine equipped with a 10 kN load cell (Figure 1b). A fixed displacement rate of 5 mm/min was applied until the rupture point. Under these conditions, four replicates per condition were planned. Tensile testing was observed using a high-speed camera (Phantom V7.3 from Photonline, Marly Le Roi, France). The deformation and rupture behavior of the samples were recorded at various frame rates, pixel sizes, and resolutions, ranging typically from (100 fps—frames per second—109 µm, 800 × 600 pixels) to (55,000 fps, 139 µm, 96 × 304 pixels). Engineering parameters, including Young’s modulus, yield stress, tensile strength, and elongation at break, were established, and the outcomes were analyzed in relation to the processing conditions. 

The X-ray microtomography technique (ESRF beamline BM5, Grenoble, France) was employed for the 3D imaging of acrylate-modified PLA/lignin samples. The acquisition parameters included 97 kV energy, 360° scan range, 1396 mm working distance, 5000 radiographic images, 101 reference images, 100 dark images, 2048 × 800 pixels detector resolution, 0.015 s count time, 0.0015 s latency time, and 3.04 µm voxel size. A back-projection reconstruction algorithm was used to acquire the tomograms. Paganin filtering was added to optimize phase retrieval of phase contrast images. In order to maintain sufficient resolution at the prescribed voxel size, two successive acquisitions along the height of the sample were conducted, allowing the acquired volume to double. The typical tomogram resolution was thus 3856 × 3856 × 1600 voxels, covering an acquired volume of 11.72 × 11.72 × 4.86 mm^3^. Image analysis was carried out using ImageJ software version 1.54 from NIH (Bethesda, MD, USA).

Part of the image processing included image brightness calibration, conversion to 256 grey level images, segmentation, 3D rotation, filtering using opening and closing operators, background elimination, phase content, anisotropy, and axial porosity level calculations.

SEM characterization was carried out on 3D-printed samples to investigate the effect of lignin addition on the quality of the prints. The observations were made using a JEOL JSM-5800LV device (JEOL-LTD, Tokyo, Japan) at a 15 KV accelerating voltage. To ensure conductivity during observation, the surfaces of the samples were coated with a 50 nm layer of carbon using a Balzers CED 30 carbon evaporator (OC Oerlikon, Balzers, Liechtenstein). Magnifications were adjusted to 90×, with typical pixel sizes ranging of 0.9 µm.

## 3. Results and Discussion

### 3.1. Chemical Structure of the Photosensitive Resins 

**Figure 2:**
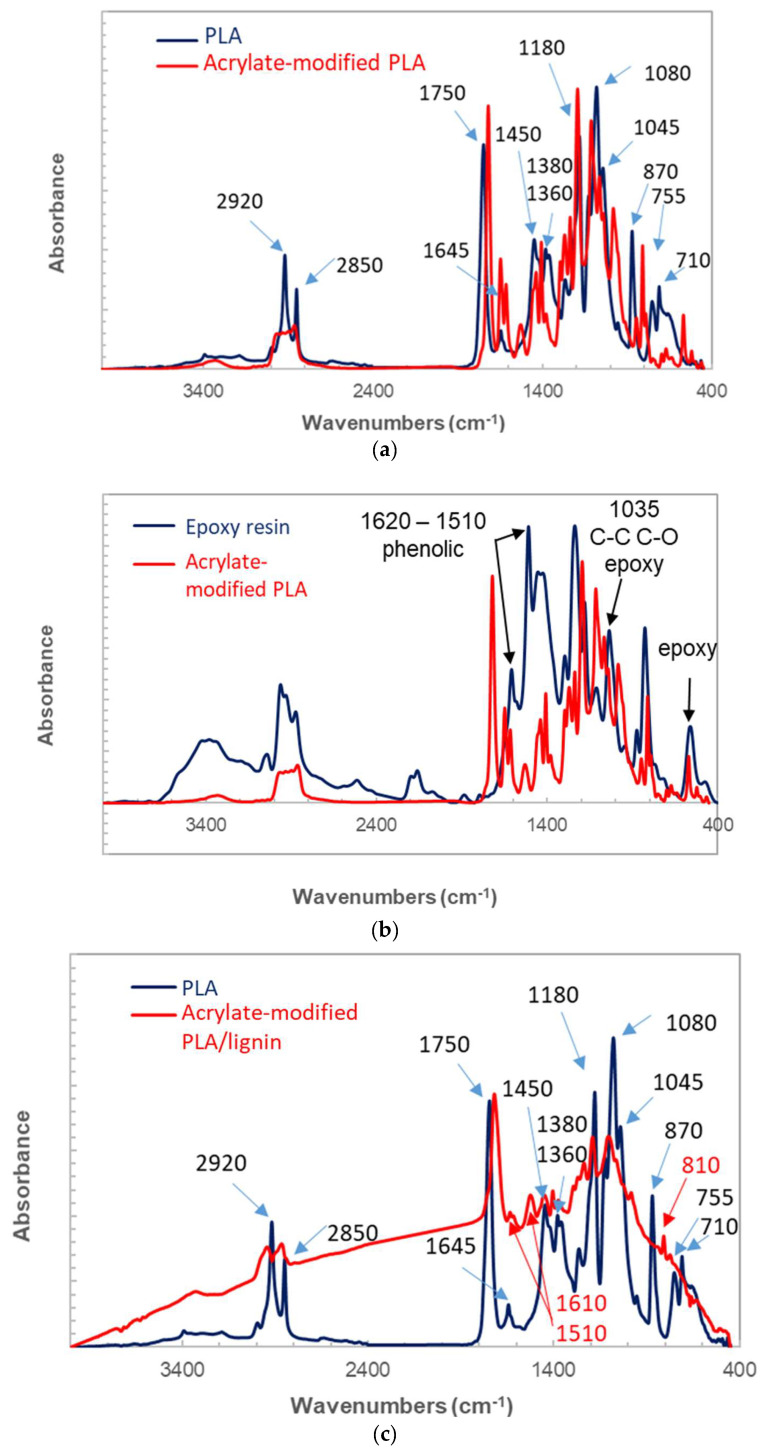
Infrared spectra of the photosensitive resins along with band width assignment: (**a**) acrylate-modified PLA resin in its liquid state compared to PLA, (**b**) acrylate-modified PLA resin in its liquid state compared to epoxy resin, (**c**) a comparison between the spectra of the acrylate-modified PLA/lignin with PLA after photopolymerization in the solid state.

Figure 2 illustrates the infrared spectra of the photosensitive acrylate-modified PLA in its liquid form and after undergoing polymerization with lignin utilized as filler. In Figure 2a, the spectral bands are assigned for the photosensitive resin in its initial state. Examination of bending and stretching frequencies reveals shared elements found in photosensitive resins, typically comprising a polymeric binder like polybutadiene, a polymerizable unsaturated monomer such as ethylhexyl acrylate, a polymerization initiator like benzoin alkyl ether or benzoin, a thermal polymerization inhibitor such as hydroquinone, and occasionally pigments. Predominantly, the key constituents within the acrylate-modified PLA resin are acrylic compounds, notably butyl-, ethyl-, and methyl-acrylate, with varying proportions. These constituents are found to be predominant in several modified photosensitive resins such as acrylated natural rubbers [40].

The distinctive infrared features assigned to acrylic compounds include the ester C=O band at 1720 cm^−1^, the ethylenic stretching C=C vibration at 1640 cm^−1^, the CH_2_ deformation at 1460 cm^−1^, and the stretching C-O band at 1190 cm^−1^. Additionally, the associated peaks at 982 and 810 cm^−1^ are attributed to the in- and out-of-plane C-H bending. In the same figure, the FTIR spectrum of PLA shows that characteristic peaks of PLA in the ranges 3300–3700 cm^−1^, 1700–1760 cm^−1^, and 500–1500 cm^−1^ appeared in both polymers [19]. These peaks correspond to both stretching and bending frequencies such as C=O, –CH_3_, and C–O [41]. 

When comparing the IR spectrum of acrylate-modified PLA with that of an epoxy resin (2-(chloromethyl)oxirane;4-[2-(4-hydroxyphenyl)propan-2-yl]phenol;prop-2-enoic acid), a better matching is achieved. The main detected peaks are aromatic C=C stretching vibration bands, C-O and C-C stretching bands, as well as those according to the frequencies shown in Figure 2b. After curing, the analysis of the IR spectra of the photosensitive acrylate-modified PLA blended with lignin is shown in Figure 2c. The PLA spectrum is also plotted as a reference material. In addition to the assignment identified in the solid state, frequencies associated with phenolic groups are also identified, namely at 1610 cm^−1^, 1510 cm^−1^, and 810 cm^−1^. 

### 3.2. Three-Dimensional Microstructure of Acrylate-Modified PLA/Lignin Blends 

**Figure 3:**
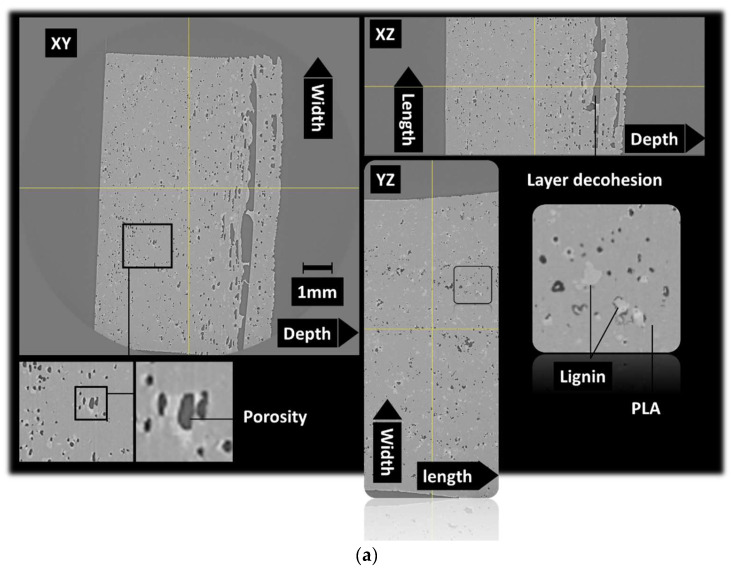

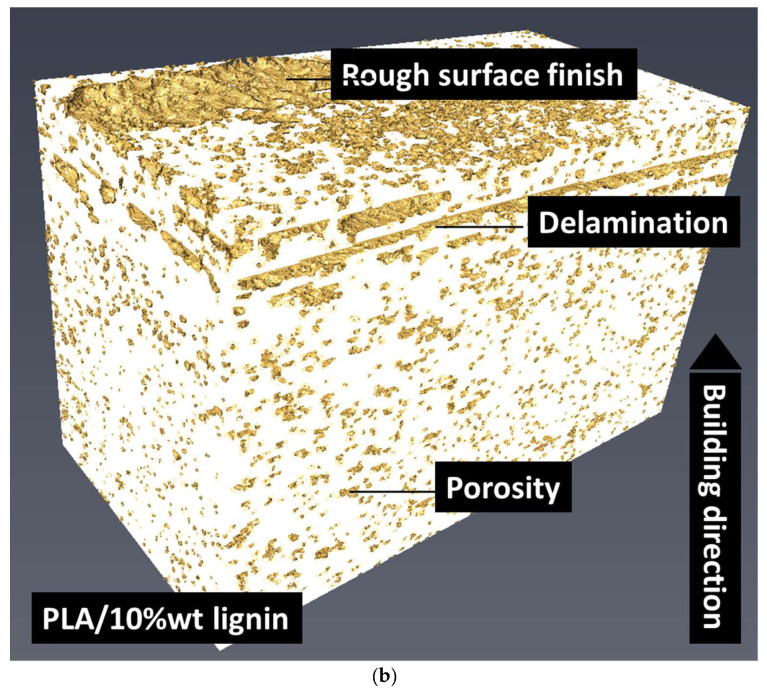
X-ray microtomography results: (**a**) orthogonal views underlying the microstructure of 3D-printed cured acrylate-modified PLA/10 wt% lignin, (**b**) 3D view of the porous structure.

The microstructure induced by the presence of lignin as a solid filler in photosensitive resins is discussed. It has to be mentioned that chemically modified lignin is generally used through its phenolic hydroxyl groups to achieve photosensitive lignin resins via the epoxidation process [42,43]. The induced microstructure is thus different from the effect of a solid filler, as demonstrated by X-ray microtomography observations. Figure 3 shows orthogonal views of an acrylate-modified PLA/10 wt% lignin blend. These views are achieved by stitching two successive tomograms taken along the height of the specimen. The region of interest has typical dimensions of 4.7 × 10 × 6.18 mm^3^. The microstructure highlights the main features related to the blending of a large amount of lignin with photosensitive acrylate-modified PLA. One of these features is the presence of porosity. The view along the XY plane shows severe delamination due to the UV blockage during the photopolymerization process. After conversion to an 8-bit image, the result of the segmentation using a grey level threshold of 138 shows the porous structure shown in Figure 3b. The 3D view demonstrates the presence of severe delamination due to the clustering of lignin during the photosensitive acrylate-modified PLA layer construction. The porosity generated by the DLP process can be attributed to the bubbles trapped by lignin incorporation. It is thus expected that the amount of porosity increases with the increase in the lignin load in the blend. Lowering of the lignin content would result in a better UV light penetration and lower extent of the delamination process. Despite the process-induced porosity, Figure 3 does not show evidence of the layering effect, as also observed in other AM technologies such as FDM. Such an effect is observed for microstructures induced by the DLP process using the same X-ray microtomography observation technique [44]. 

**Figure 4:**
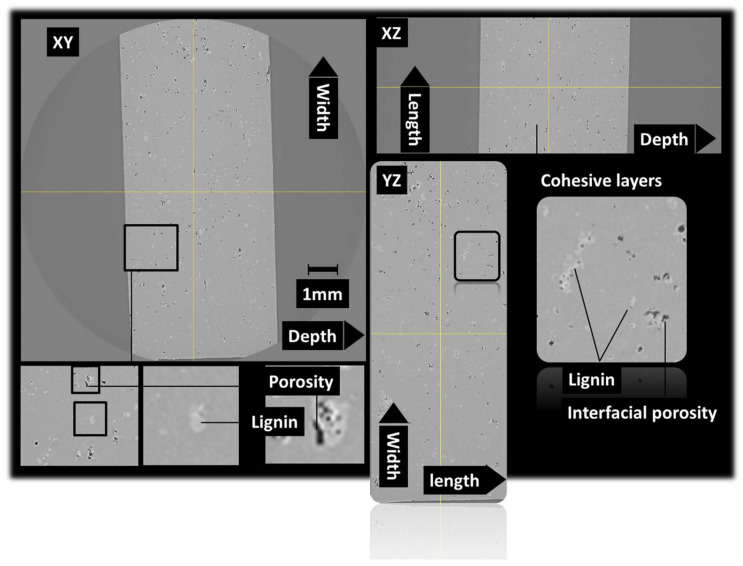
X-ray microtomography results: orthogonal views underlying the microstructure of 3D-printed cured acrylate-modified PLA/5 wt% lignin.

Figure 4 shows the same orthogonal views for the acrylate-modified PLA sample blended with 5 wt% of lignin. The same mesoscopic porosity can be found but to a lesser extent compared to Figure 3. As expected, the delamination process also occurred as a direct result of the improvement of the photopolymerization process. A particular feature can be also read from the analysis of Figure 4, which is that the presence of porosity is spatially related to the presence of lignin. Indeed, this porosity can be classified as interfacial porosity. It can be thus deduced that the process-generated porosity is correlated to the photopolymerization process blockage induced by some shadowing effect of lignin. 

**Figure 5:**
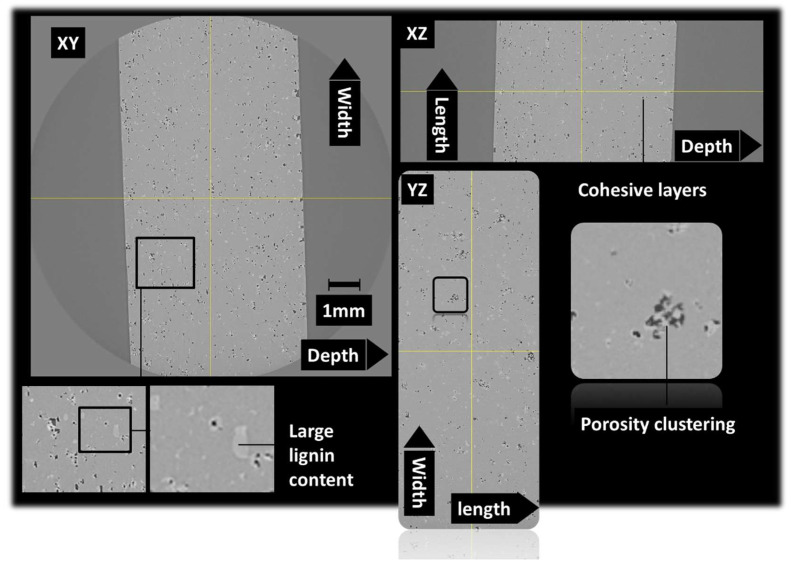
X-ray microtomography results: orthogonal views underlying the microstructure of 3D-printed cured acrylate-modified PLA /10 wt% lignin with an intense mixing stage.

In an attempt to improve the lignin content, an intense mixing stage was added prior the photopolymerization process. The duration of this mixing phase was extended by an extra 5 min compared to the initial mixing. Following this, photopolymerization occurred immediately without any delay. The result of microstructural analysis is shown in Figure 5. This figure highlights a slightly larger porosity content compared to the case of acrylate-modified PLA blended with 5 wt% of lignin. Nevertheless, the outcome of the intense mixing is indisputable. The additional mixing before the photopolymerization process eliminates layer delamination and reduces the level of porosity while maintaining a larger lignin content. 

**Figure 6:**
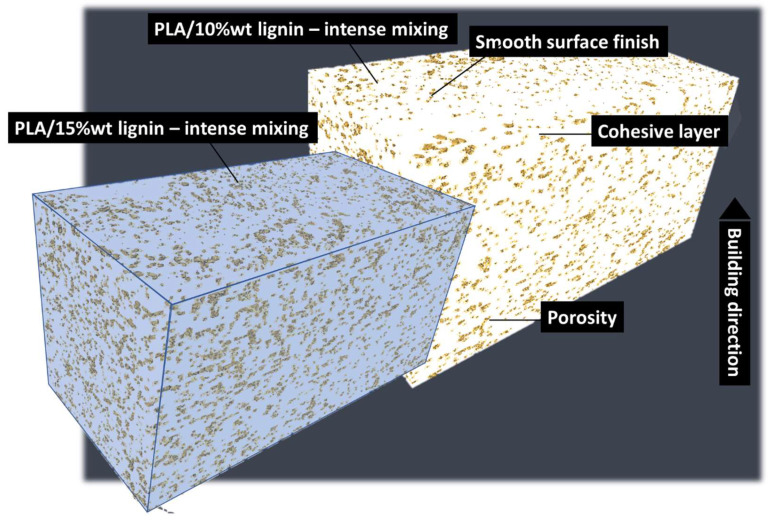
X-ray microtomography results: comparison between the microstructural features of 3D-printed cured acrylate-modified PLA/10 wt% and 15 wt% lignin.

The same intense mixing stage was considered for even larger lignin content of 15 wt%. Figure 6 shows the result of both acrylate-modified PLA blends with 10 wt% lignin and 15 wt% lignin microstructures illustrated through the perspective views. There is no delamination detected with a 15 wt% blending of lignin, but there is an observed increase in porosity clustering. This clustering appears to also promote pore connectivity perpendicular to the construction direction.

Further analysis was conducted to quantify the anisotropic spatial distribution of the process-generated porosity, as well as the shape and size distribution. 

The axial porosity profiles were explored for all considered formulations. These profiles were obtained by counting the black voxels belonging to the porosity phase in a particular direction normalized with respect to the length. This was as follows for the porosity profiles in the depth direction:

(1) $$f_{j}\left(\%\right)=\sum_{i,k=1}^{N_{X}\times N_{Z}}\left(1-\left(g_{ijk}/255\right)\right)/\left(N_{X}\times N_{Z}\right)$$

where $f_{j}$ is the porosity level in the Y-direction at the jth position, i.e., along the depth or construction direction, $N_{X}$, $N_{Z}$ are the total number of voxels in the X- and Z-directions, and correspond to the height of the specimen. $g_{ijk}$ is the grey level associated with the voxel at the coordinates *i*,*j*,*k*. For each voxel, the grey level is either 255 corresponding to the solid phase (lignin or acrylate-modified PLA in the segmented image), or 0 for porosity. 

**Figure 7:**
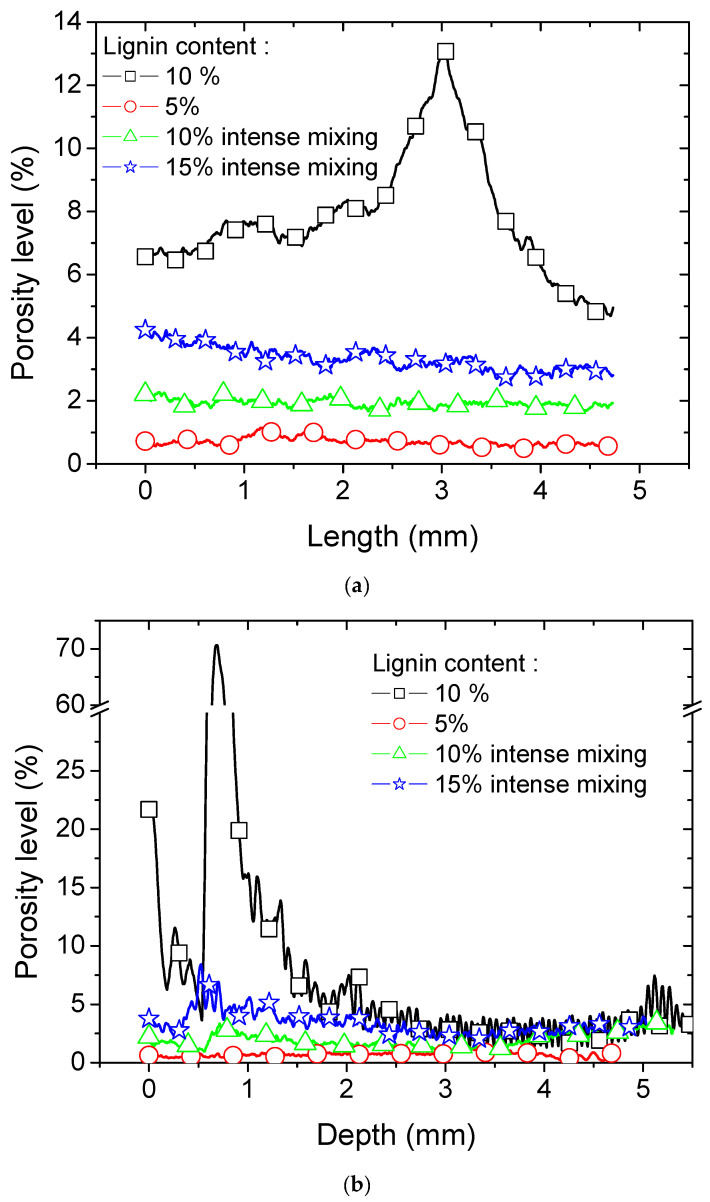

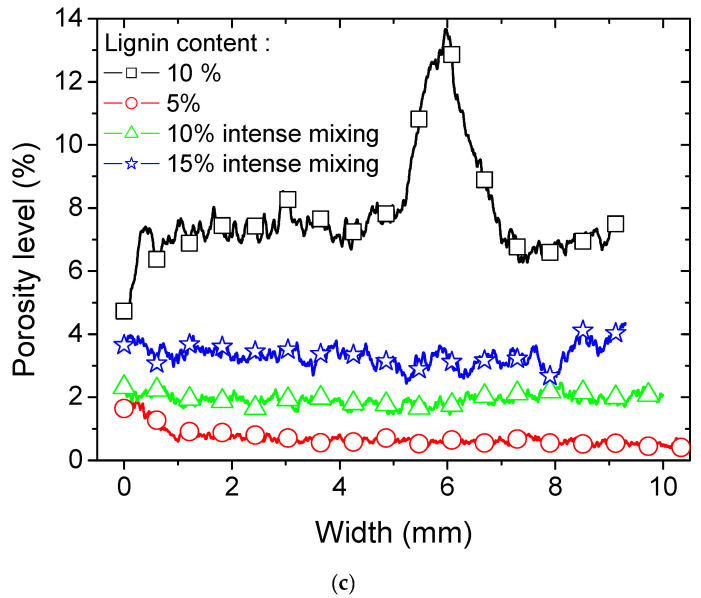
Axial porosity level profiles along (**a**) the length, (**b**) construction (depth) direction, and (**c**) width direction.

Figure 7a shows the result of the porosity profiles along the length direction. A large peak in the porosity level culminating at nearly 13% is observed for the acrylate-modified PLA/10 wt% lignin sample. This porosity peak is associated with the delamination process occurring for this case without considering the intense mixing stage. The average porosity level that can be extracted from this profile is close to 7.82%. Acrylate-modified PLA blended with 5 wt% of lignin shows a nearly flat profile with slight jaggedness that can be attributed to the layering effect. The average porosity level is only 0.71%, which is more in line with the type of porosity generated from the DLP process. With an intense mixing stage, the same blend of 10 wt% lignin shows an eventually higher porosity level along the length, but its trend is closer to that of the acrylate-modified PLA/lignin 5 wt% blend. It lacks notable fluctuations in porosity levels throughout the length, and more crucially, it does not exhibit the same porosity peak as observed in the case of the acrylate-modified PLA/lignin 10 wt% blend without an intensive mixing stage, indicating the absence of delamination. The average porosity level in this case is 1.92%, which is closer to the peak value of 2.28% observed for the same blend. The highest lignin content of 15 wt% does generate a higher porosity of about 3.33%, but there is no peak value, which indicates the presence of inter-layer delamination. The only difference in this case with respect to lower contents is the presence of a gradient in the porosity level, which can be attributed to the less cohesive structure close to the curvature region. 

Figure 7b shows the result of the porosity profiles along the depth direction. This direction corresponds to the building direction. The same ranking of porosity levels is depicted, where the best performing blend is acrylate-modified PLA/lignin 5 wt% and the worst is acrylate-modified PLA/lignin 10 wt% without an intense mixing stage. All conditions share a common characteristic: the porosity level profile along the depth direction can be identified through the consistent variation in porosity levels, as observed in the zoomed-in view. The distance between peaks corresponds to a layer height of 50 µm. This regular variation is particularly noticeable in the acrylate-modified PLA/lignin 10 wt% blend. Another notable aspect is the asymmetry in porosity levels at the top and bottom depths. Specifically, a significant porosity level is linked to the initial built layers before reaching a stable variation. This asymmetry can be attributed to varying UV exposure conditions at the bottom of the specimen, where the blend adheres to the platform. Importantly, it should be noted that the impact of these specific exposure conditions extends well beyond the initial five layers affected by intense UV exposure. The depth affected by these conditions is approximately 250 µm, and the disturbance in porosity levels extends to the first 1.5 mm of the sample depth.

Figure 7c shows the result of the porosity profiles along the width direction. These profiles confirm the same trends achieved for both length and depth directions. More homogeneous porosity levels are obtained for most blends, with the exception of the acrylate-modified PLA/lignin 10 wt% blend due to the lack of mixing, which directly affects the homogeneity of lignin spatial distribution. Although the blend formed with 15 wt% of lignin looks promising, it has limited reproducibility for prints as large as 100 mm. A sufficient number of dog-bone specimens could not be achieved to conduct the tensile performance analysis. In the following, only the 5 wt% and 10 wt% lignin blends are compared with respect to the green and post-curing states. 

### 3.3. Mechanical Results

**Figure 8:**
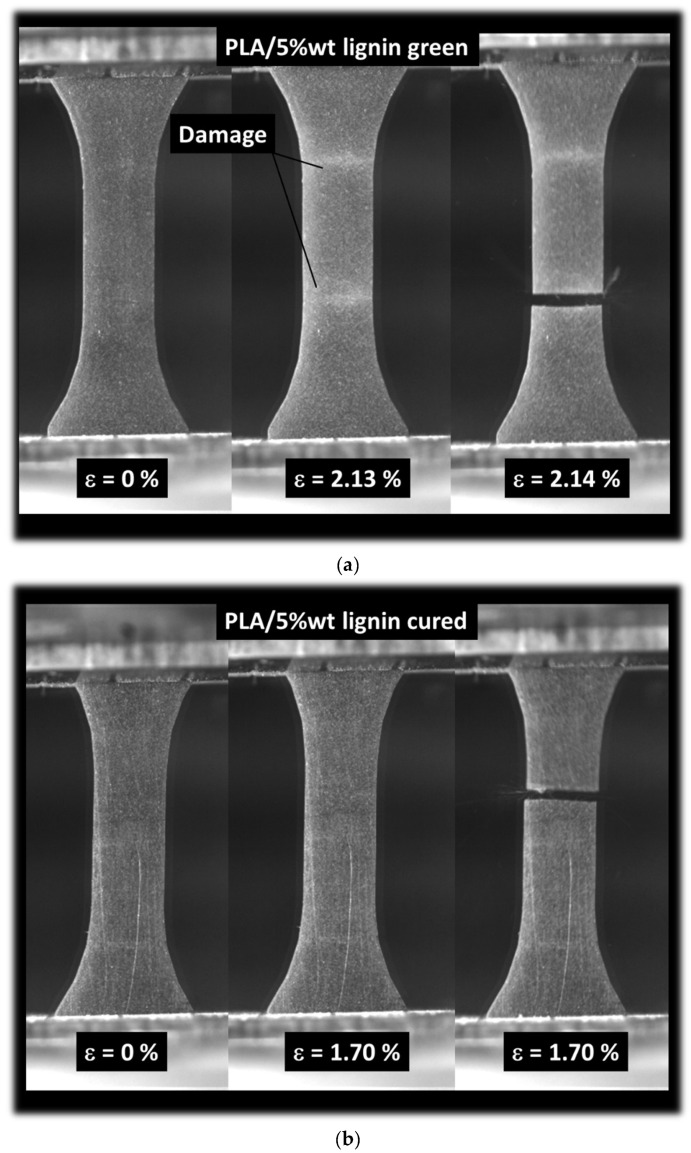
Summary of the tensile results, optical recording: (**a**) acrylate-modified PLA/lignin 5 wt% green blend, (**b**) acrylate-modified PLA/lignin 5 wt% cured blend.

Figure 8 illustrates the characteristic tensile response of 3D-printed acrylate-modified PLA/lignin 5 wt% specimens with or without a post-curing stage. In Figure 8a, the green sample exhibits a quasi-brittle behavior. Before the material undergoes rupture, there are indications of cumulative damage, manifested as whitening in the direction perpendicular to the loading direction. The rupture seems to occur through transverse cracking without a significant deviation in the main crack. It can be asserted that the predominant failure mechanism involves the opening mode. Figure 8b shows the homologue situation for the cured sample. No remarkable difference is observed, with the exception of a reduction in the stretching prior to rupture. 

**Figure 9:**
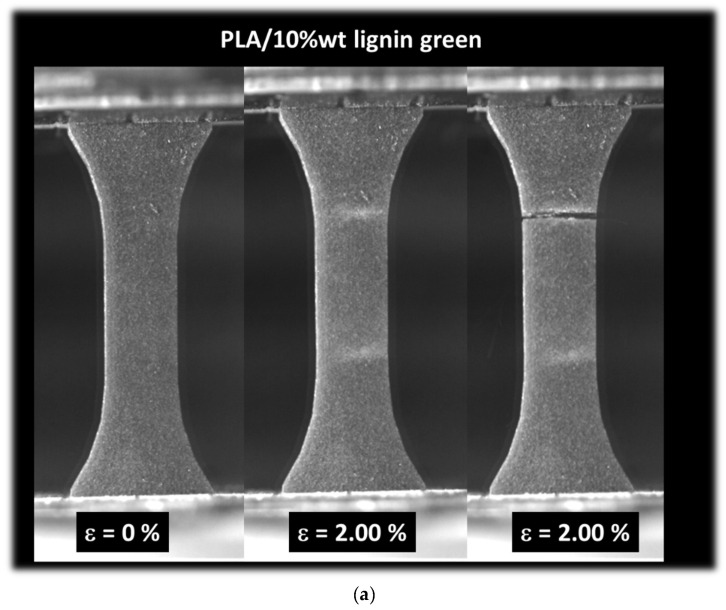

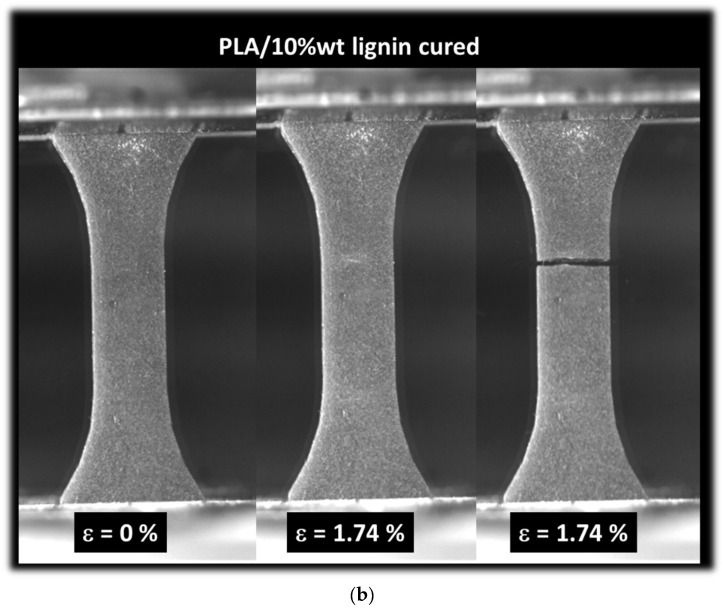
Summary of the tensile results, optical recording: (**a**) acrylate-modified PLA/lignin 10 wt% green blend, (**b**) acrylate-modified PLA/lignin 10 wt% cured blend.

Figure 9 depicts the characteristic tensile behavior of 3D-printed acrylate-modified PLA/lignin 10 wt% specimens, with or without a post-curing stage. In Figure 9a, the green sample displays a quasi-brittle behavior similar to that observed in the acrylate-modified PLA/lignin 5 wt% blend. However, the extent of damage accumulated before rupture appears more pronounced in this case, likely attributed to the higher concentration of porosities serving as stress concentrators. The stretching behavior seems to be approximately the same as that observed in the acrylate-modified PLA/lignin 5 wt% blend, and the rupture of the material follows a similar opening mode. In Figure 9b, the situation for the cured sample is shown. No significant difference is observed, except for a reduction in stretching before rupture. The quasi-brittle behavior observed in Figure 9a is similar to the behavior of 3D-printed biobased composites engineered using fused filament fabrication. Indeed, a former study relates the predominant opening mode to the brittleness of the material [45]. In the present case, such brittleness is caused by the UV curing process [40]. In the case of fused filament fabrication, the brittleness is genuine to the filament orientation combined the feedstock material intrinsic performance [46]. 

**Figure 10:**
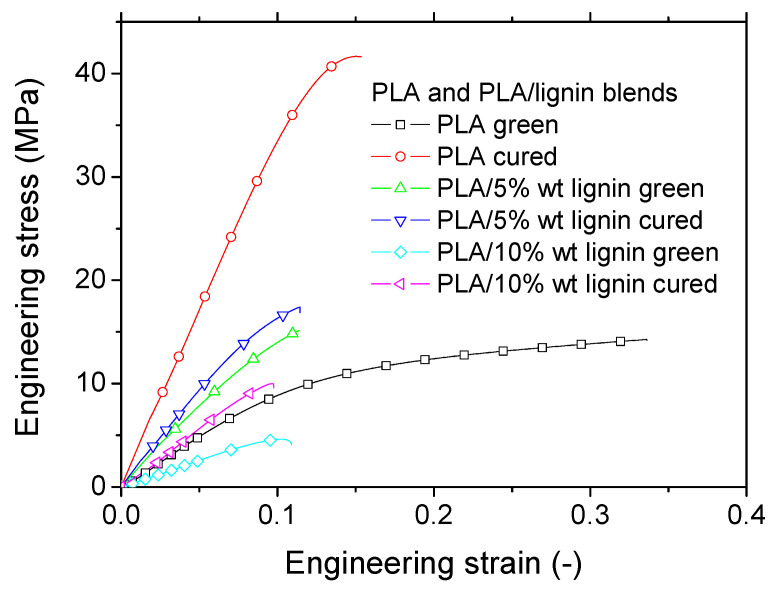
Tensile performance of acrylate-modified PLA and acrylate-modified PLA/lignin blends as a function of post-curing exposure.

The tensile response of all studied conditions is further shown in Figure 10 considering the engineering strain–stress curves. All samples exhibit elastic–plastic behavior, where the extent of the plasticity stage is primarily related to the presence of a post-curing stage. Indeed, green samples exhibit a larger plasticity stage compared to the cured samples. The addition of lignin seems to lower the mechanical response more significantly when the amount of lignin is large. This is attributed to the presence of porosity induced by the processing. More quantitative analysis of lignin addition is summarized in Table 2. Engineering constants such as Young’s modulus (E_Y_), yield stress (σ_Y_), relative width of plastic stage (W), tensile strength (σ_T_), and elongation at break (ε_R_) were extracted from the tensile responses of at least three replicates. It is important to note that the reproducibility of the results outlined in Table 2 depends on the consistency of lignin distribution within the photosensitive resin, which is influenced by its weight percentage. The maximum deviation observed for tensile strength and stiffness is 18% and 22%, respectively, when the lignin weight content is at its highest (10%). This deviation reduces to 4% when the lignin content is 5 wt%.

According to Table 2, the UV curing does not affect the density of the studied 3D material. However, the addition of lignin increases the density of the 3D-printed blend. Indeed, the density of lignin (1.35 to 1.50 g/cm^3^) [47] is higher than that of PLA (1.11 g/cm^3^) by 20% to 35%. A linear fitting performed on data in Table 2 shows the following trend:

(2) $$\rho_{B}\left(g/{cm}^{3}\right)=1.11+0.06\times \rho_{P}\left(g/{cm}^{3}\right)\;\;\;\;\;\;\;\;R^{2}=0.98$$

where $\rho_{B}$ and $\rho_{P}$ are the density of the blend and PLA, respectively, and R² is the correlation factor. 

In its green state, the yield stress of the blend is found to be negatively affected by the addition of lignin:

(3) $$\sigma_{YB}\left(MPa\right)=8.68-0.34\times \sigma_{YP}\left(MPa\right)\;\;\;\;\;\;\;\;R^{2}=0.94$$

where $\sigma_{YB}$ and $\sigma_{YP}\;$ are the yield stress of the blend and PLA, respectively.

In the cured state, the same correlation if found but according to a nonlinear trend and a higher intensity. The linear approximation shows the following trend:

(4) $$\sigma_{YB}\left(MPa\right)=31-3.5\times \sigma_{YP}\left(MPa\right)\;\;\;\;\;\;\;\;R^{2}=0.89$$

The decrease in the yield stress can be explained by the increasing stiffness and brittleness of the 3D-printed blend. 

The stiffness test results reveal a threefold enhancement in the original stiffness of untreated acrylate-modified PLA when post-curing is applied. However, this ratio decreases to 1.36 when lignin is introduced to acrylate-modified PLA. The stiffness of the acrylate-modified PLA blend experiences an average reduction of 45% with the addition of 5 wt% of lignin. This reduction becomes even more substantial with 10 wt% of lignin (68%).

In comparison to this decrease in stiffness, the density of the blend is 1.32 to 1.52 times greater than the density of pure acrylate-modified PLA.

The yield stress also increases in the case of cured samples, and the width of the plasticity stage, expressed as the total strain beyond the yield strain up to the rupture, decreases. 

In order to provide further insights into the acceptable lignin content that contributes positively to the environmental footprint and cost reduction while limiting the loss in performance, the following equations are achieved representing the correlation between lignin content, stiffness, and tensile strength. These correlations are provided based on simple linear fitting routines using data in Table 1 according to blends in the cured state:

(5) $$E_{Y}\left(MPa\right)=346-25\times f_{l}\left(\%\right)\;\;\;\;\;\;\;\;R^{2}=0.92$$

and

(6) $$\sigma_{T}\left(MPa\right)=43-3.7\times f_{l}\left(\%\right)\;\;\;\;\;\;\;\;R^{2}=0.83$$

where *E_Y_* is Young’s modulus, σ_T_ is tensile strength, $f_{l}$ is the weight content of lignin in the blend, and R² is the correlation factor. 

Even if the linear correlation seems to have a limited validity in the case of the tensile strength, Equations (5) and (6) provide an approximation of acceptable loss in mechanical performance when lignin is used in the photosensitive material. Taking a 22% reduction in the mechanical performance as a threshold, which is within the standard deviation of experimental results, the optimal lignin content that can be used without significantly affecting the performance is 3%.

Table 3 compares some of the lignin-based blends using the epoxidization/UV curing route similar to the DLP process used in this study. Although it is difficult to compare the performances due to the varied matrices and composites attempted, Table 3 still shows some key mechanical performance indicators for the use of lignin as a blend in 3D-printing processes. For instance, Yan et al. [48] considered organosolv lignin-based epoxy acrylate films. The developed gels exhibited high hardness for intermediate lignin contents of 5% and 10% compared to the blends prepared using 2% of lignin. 

Nisha et al. [49] developed ionic liquid lignin epoxy composites with up to 2wt% of lignin mixed with a prepolymer epoxy resin and an anhydride-based curing agent. Among the mechanical tests, the authors considered both tensile and flexural tests, achieving a positive effect from adding 2wt% of lignin. Typical tensile strengths achieved were around 40 MPa. Zhen et al. [50] developed lignin-based epoxy resin using the epoxidization route. The authors demonstrated a link between the cross-link density and improved flexural and impact performances. Arias-Ferreiro et al. [51] considered the DLP route to achieve 3D printing of lignin/pTSA-PANI/acrylic composite. The authors showed that the printability of the composites decreased with lignin content as large as 3wt%. The same authors showed that tensile performance, especially tensile strength (<2 MPa), is affected by the imperfect curing caused by lignin aggregation. Zhang et al. [52] obtained 3D-printed lignin-reinforced composites using stereolithography. With the small addition of lignin in the photopolymerized composite, the authors showed that lignin content positively affects the tensile strength (from 30 to 50 MPa) after post-curing. 

### 3.4. SEM Analysis of Ruptured Samples 

**Figure 11:**
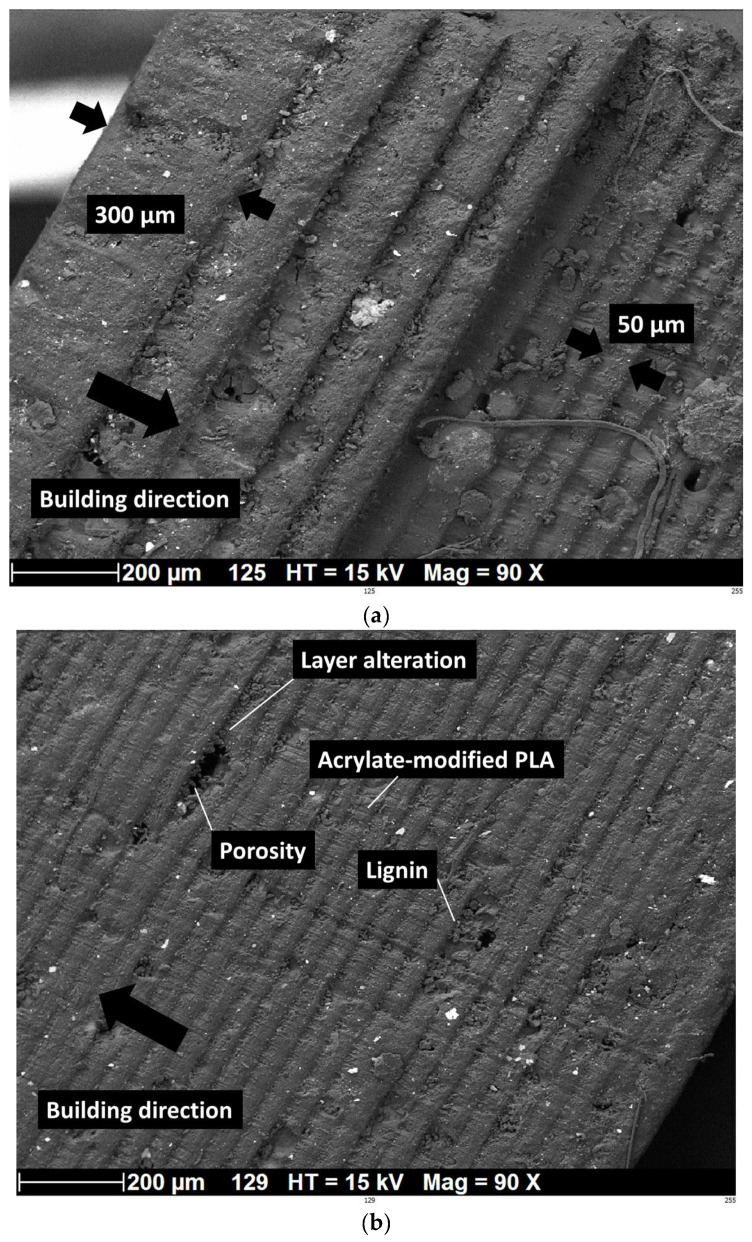
SEM analysis of 3D-printed acrylate-modified PLA/lignin 5 wt% in the cured state: (**a**) zoomed-in view across the depth showing first built layers, (**b**) zoomed-out view of the laying arrangement. Black arrows indicate the building direction and the thickness of built layers.

The microstructures of the fractured samples were analyzed using scanning electron microscopy (SEM) to further investigate the reasons behind the reduced mechanical properties of printed parts containing lignin compared to pure PLA. Figure 11a shows the building characteristics of acrylate modified with the addition of 5 wt% of lignin in the green state. This zoomed-in view normal to the building direction shows the specific printing conditions associated with the first five layers. Since the addition of lignin affects the DLP printability, specifically the cure depth, as well as the cross-linking degree of the printed part, specific printing settings are associated with the first layers in contact with the building platform. The layer height decreases from 300 µm to 50 µm to ensure cohesion between the platform and the specimen. In addition, the exposure time is increased to 20 s for these layers. Figure 11b shows a zoomed-out view, allowing a better evaluation of the effect of lignin on the quality of the prints. With the same 5% of lignin added to acrylate-modified PLA, the alteration in the laying is observed, as well as the presence of porosity left by the removal of lignin during processing. The observed layering effect from SEM micrographs confirms the X-ray microtomography results [44]. 

**Figure 12:**
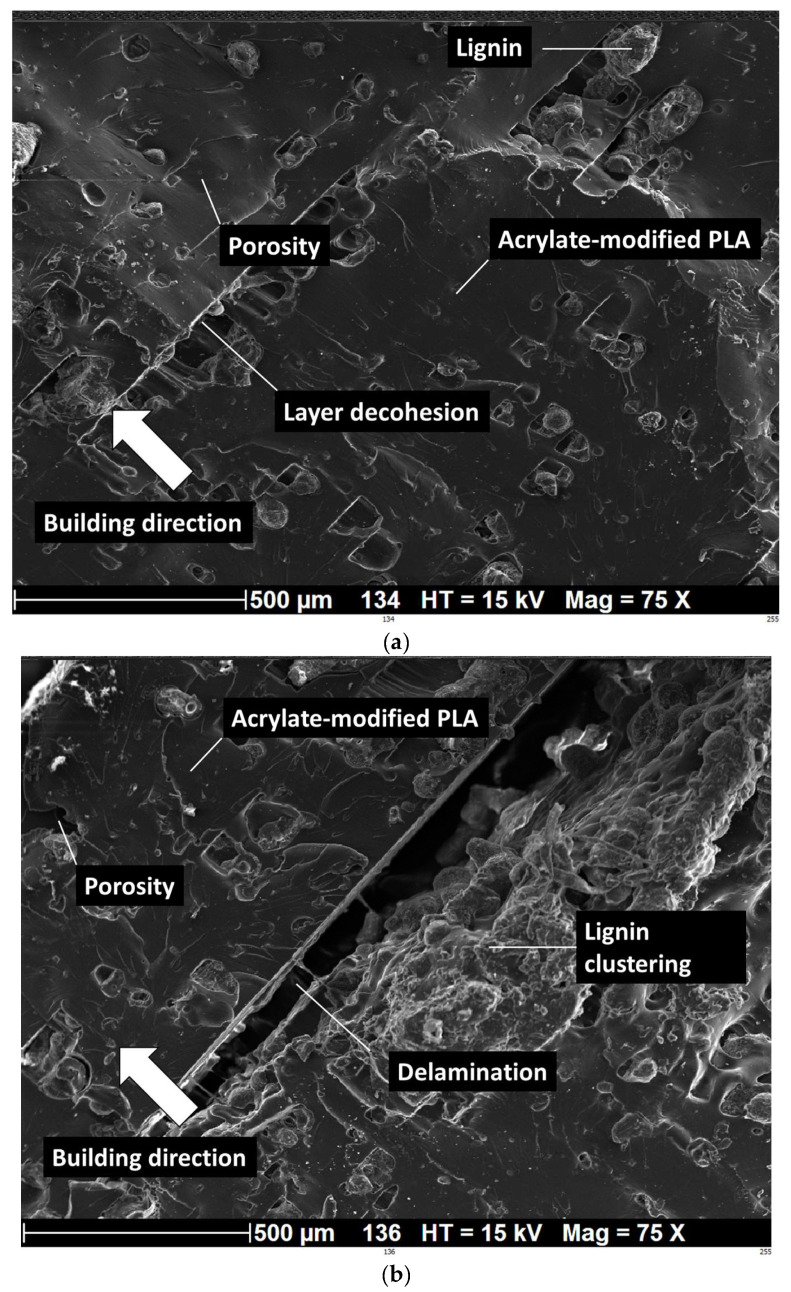
SEM analysis of 3D-printed acrylate-modified PLA/lignin 10 wt% in the cured state: (**a**) zoomed-in view across the depth showing layer decohesion due to lignin clustering, (**b**) zoomed-out view of the laying arrangement. White arrows indicate the building direction.

Figure 12 exhibits the same type of micrographs for a larger lignin content of 10%. The zoomed-in view in Figure 12a demonstrates well the clustering effect on the quality of the print. This is materialized by the decohesion between adjacent layers as a consequence of the UV blockage. The lignin clustering eventually leads to delamination, as shown in Figure 12b and confirmed by the X-ray microtomography observations in Figure 3. This effect of rigid lignin microparticles blocking UV curing was also observed by Arias-Ferreiro et al. [51] in the case of lignin/pTSA-PANI/acrylic composite obtained by DLP. 

**Figure 13:**
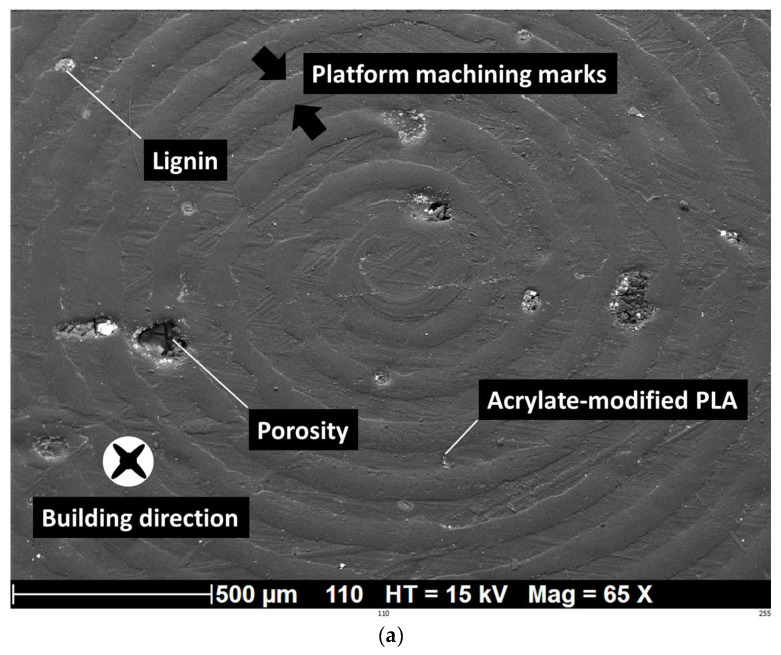

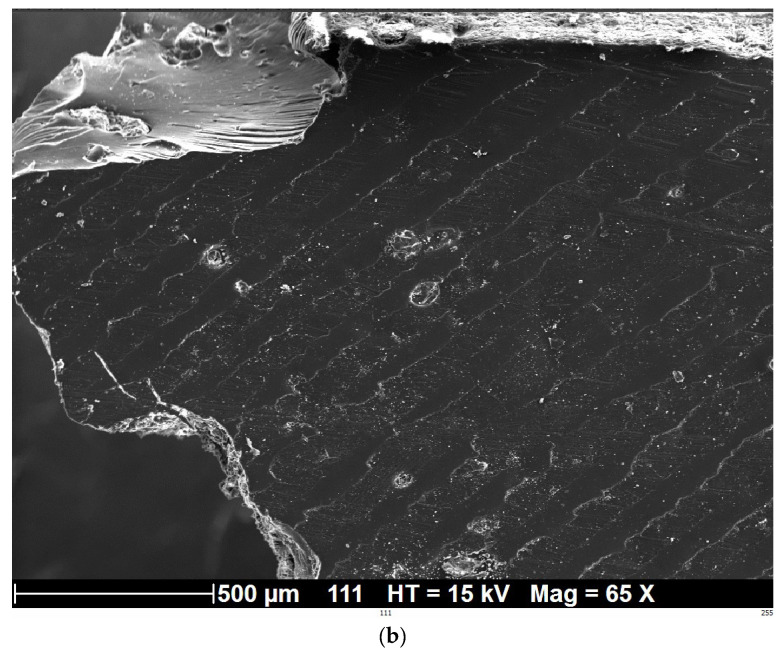
SEM analysis of 3D-printed acrylate-modified PLA/lignin 5 wt% in the cured state: (**a**) view normal to the building direction, (**b**) lateral view showing crack pattern. Arrows indicates the marks of the machining process.

In addition to the lignin effect on the layering of prints, Figure 13a shows the effect of the surface finish of the building platform on the generated roughness on the specimens. The rings produced by the machining of the metallic building platform generate typical roughness on the bottom surface of the specimens. The typical width of these rings is 70 µm and the spacing is close to 80 µm. The lignin presence also modifies the surface finish by creating multiple craters, which negatively contribute to the adhesion between the printing platform and the printed material. This effect seems limited, especially for lignin weight contents as small as 5%. Figure 13b shows a lateral view of the cracking pattern. This micrograph confirms the quasi-brittle nature of the failure of the material as well as the slight deviation of the main crack with respect to the transverse direction, as discussed from optimal imaging (Figure 8). 

## 4. Conclusions

This investigation underscores the potential of incorporating lignin as a filler in a photopolymerization process with acrylate-modified PLA to reduce feedstock costs and enhance the environmental sustainability of technical components produced through additive manufacturing. DLP technology, in contrast to other methods such as fused filament deposition, eliminates the need for processing lignin at high temperature, addressing a significant challenge of lignin degradation. Successful trials have been conducted using DLP to 3D print samples incorporating a 10% lignin filler. The combination of lignin content and post-curing led to improved tensile properties, although the acrylate-modified PLA/lignin blends rank lower than pure acrylate-modified PLA in terms of tensile response. The observed tensile performance is primarily influenced by the level of process-induced porosity resulting from the UV blockage caused by lignin addition. All blends exhibit elastic–plastic behavior, where the width of the plasticity stage is inversely correlated with a higher amount of lignin and post-curing. This study demonstrates that lignin amounts of approximately 5 wt% do not impact the photopolymerization process, regardless of the curing stage. In addition, this study concludes that the linear decrease in tensile strength and stiffness with respect to lignin content can be considered to be limited for weight contents of lignin as small as 3%. As future work, the granulometry of lignin powder and the premixing stage are possible parameters that could be used to improve the maximum content of lignin in photosensitive resins without altering the tensile performance. These parameters will be considered in a future work to achieve a better substitution rate of the synthetic feedstock materials in DLP. 

## Figures and Tables

**Table 1:** Key attributes of the photosensitive acrylate-modified PLA resin.

Property	Magnitude	Property	Magnitude
Viscosity (25 °C, MPa.s)	138 ± 18	Flexural strength	59 ± 18 MPa
Wavelength (nm)	400 ± 7	Flexural modulus	1.07 ± 0.10 GPa
Density (g/cm^3^)	1.11 ± 0.04	Hardness score	81 ± 1 (shore D)
Tensile strength (MPa)	46.50 ± 0.71	IZOD impact strength	29 ± 16 J/m
Elongation at break (%)	31.5 ± 4.95	-	-

**Table 2:** Mechanical performance of 3D-printed acrylate-modified PLA and acrylate-modified PLA/lignin blends as a function of UV post-exposure. ρ density, E_Y_: Young’s modulus, σ_Y_: yield stress, W_P_: Relative width of plastic stage, σ_T_: tensile strength, ε_R_: elongation at break.

Material	UV	ρ (g/cm^3^)	E_Y_ (MPa)	σ_Y_ (MPa)	W_P_ (%)	σ_T_ (MPa)	ε_R_ (−)
Acrylate-modified PLA	Green	1.11 ± 0.00	114 ± 14	8.5 ± 1.8	81 ± 4.3	16.50 ± 2.0	0.39 ± 0.05
Acrylate-modified PLA	Cured	1.12 ± 0.00	346 ± 12	34.8 ± 1.1	35 ± 1.3	42.65 ± 2.9	0.16 ± 0.01
Acrylate-modified PLA/lignin 5 wt%	Green	1.48 ± 0.03	137 ± 17	7.7 ± 2.5	53 ± 1.4	13.38 ± 2.5	0.12 ± 0.00
Acrylate-modified PLA/lignin 5 wt%	Cured	1.47 ± 0.03	188 ± 6	11.8 ± 0.6	45 ± 0.5	17.79 ± 0.7	0.11 ± 0.01
Acrylate-modified PLA/lignin 10 wt%	Green	1.71 ± 0.07	78 ± 36	5.1 ± 2.0	36 ± 0.1	7.15 ± 3.6	0.10 ± 0.01
acrylate-modified PLA/lignin 10 wt%	Cured	1.68 ± 0.02	108 ± 19	7.0 ± 1.6	32 ± 0.6	9.24 ± 2.0	0.10 ± 0.00

**Table 3:** Comparison between 3D-printed lignin blends.

Reference	Material	Route	Lignin * Content (%)	Performance
Nisha et al. [49]	Ionic liquid lignin epoxy composite	UV curing	2	Flexural, tensile
Yan et al. [48]	Organosolv lignin-based epoxy acrylate film	UV curing	25	Hardness, adhesion, and flexibility
Zhen et al. [50]	demethylated-phenolated lignin-basedepoxy resin	Epoxidization	-	Flexural, impact
Arias-Ferreiro et al. [51]	Lignin acrylic composites	DLP	4	Flexural
Zhang et al. [52]	Lignin-methacrylateresin composite	Stereolithography	1	Tensile

* maximum content.

## Data Availability

Data are available from the authors upon request due to legal reasons.
